# Early Hemoperfusion May Improve Survival of Severely Paraquat-Poisoned Patients

**DOI:** 10.1371/journal.pone.0048397

**Published:** 2012-10-29

**Authors:** Ching-Wei Hsu, Ja-Liang Lin, Dan-Tzu Lin-Tan, Kuan-Hsing Chen, Tzung-Hai Yen, Mai-Szu Wu, Shih-Chieh Lin

**Affiliations:** 1 Division of Clinical Toxicology, Department of Nephrology, Chang Gung Memorial Hospital, Lin-Kou Medical Center, Taoyuan, Taiwan, Republic of China; 2 Chang Gung University and School of Medicine, Taoyuan, Taiwan, Republic of China; 3 Department of Nephrology, Keelung Chang Gung Memorial Hospital, Keelung, Taiwan, Republic of China; Kaohsiung Chang Gung Memorial Hospital, Taiwan

## Abstract

**Background:**

Thousands of paraquat (PQ)-poisoned patients continue to die, particularly in developing countries. Although animal studies indicate that hemoperfusion (HP) within 2−4 h after intoxication effectively reduces mortality, the effect of early HP in humans remains unknown.

**Methods:**

We analyzed the records of all PQ-poisoned patients admitted to 2 hospitals between 2000 and 2009. Patients were grouped according to early or late HP and high-dose (oral cyclophosphamide [CP] and intravenous dexamethasone [DX]) or repeated pulse (intravenous methylprednisolone [MP] and CP, followed by DX and repeated MP and/or CP) PQ therapy. Early HP was defined as HP <4 h, and late HP, as HP ≥4 h after PQ ingestion. We evaluated the associations between HP <4 h, <5 h, <6 h, and <7 h after PQ ingestion and the outcomes. Demographic, clinical, laboratory, and mortality data were analyzed.

**Results:**

The study included 207 severely PQ-poisoned patients. Forward stepwise multivariate Cox hazard regression analysis showed that early HP <4 h (hazard ratio [HR] = 0.38, 95% confidence interval (CI) 0.16–0.86; *P = *0.020) or HP <5 h (HR = 0.60, 95% CI: 0.39–0.92; *P = *0.019) significantly decreased the mortality risk. Further analysis showed that early HP reduced the mortality risk only in patients treated with repeated pulse therapy (n = 136), but not high-dose therapy (n = 71). Forward stepwise multivariate Cox hazard regression analysis showed that HP <4.0 h (HR = 0.19, 95% CI: 0.05–0.79; *P = *0.022) or <5.0 h (HR = 0.49, 95% CI: 0.24–0.98; *P = *0.043) after PQ ingestion significantly decreased the mortality risk in repeated pulse therapy patients, after adjustment for relevant variables.

**Conclusion:**

The results showed that early HP after PQ exposure might be effective in reducing mortality in severely poisoned patients, particularly in those treated with repeated pulse therapy.

## Introduction

Paraquat (PQ) (1,1′-dimethyl-4,4′-bipyridium dichloride) is one of the most widely used herbicides in the world. In humans, whether intentional or accidental, ingestion of PQ is frequently fatal, causing significant lung injury [1]. Although cases of PQ poisoning are rare in developed countries, PQ poisoning remains a major cause of mortality for thousands of people in less-developed countries.

The mortality rate of acute PQ poisoning is highly correlated with plasma and urine PQ concentrations [2], [3], [4]. The sodium dithionite urine test for PQ intoxication is based on the reduction of PQ by sodium thionite at an alkaline pH to form a stable blue radical ion. A navy blue (≥25 ppm and <50 ppm) or dark blue (≥50 ppm) color detected from the urine test within 24 h after PQ ingestion generally indicates severe PQ poisoning, which is associated with poor outcome and high mortality in these patients [3], [4], [5]. A previous study [3] that used the urine PQ test as the prognostic index to assess 53 patients with severe PQ poisoning demonstrated that 36 patients with navy blue and dark blue colored urine test reactions within 24 h after PQ ingestion had an 88.9% (32/36) mortality rate. In contrast, the 17 patients with colorless or light blue colored urine test results within 24 h after PQ ingestion had a 23.5% (4/17) mortality rate. Another study [4] also demonstrated that 65 severely PQ-poisoned patients, indicated by navy blue and dark blue colored urine test results, also had an 81.5% (53/65) mortality rate, despite administration of conventional high-dose cyclophosphamide (CP) and dexamethasone (DX) treatments for PQ intoxication. Furthermore, another study [5] of blood filtration in treating PQ-poisoned patients indicated that patients with dark blue colored urine test results within 24 h following ingestion had 97% mortality rates (32/33). Taken together, these findings suggested that urine PQ tests within the first 24 h of exposure are good predictors of outcome, as navy blue and dark blue color in urine tests indicated severe PQ poisoning; colorless and light blue color in urine tests indicated mild to moderate PQ poisoning.

Hemodialysis (HD) or hemoperfusion (HP) is a comprehensive therapy administered in the initial stages of PQ intoxication. PQ clearance is apparently more effective with HP than with HD therapy [6]. In clinical practice, the causes of mortality from PQ poisoning are multiple organ failure (acute stage) and lung fibrosis-related respiratory failure (sub-acute stage) [1]. Although HP can effectively remove PQ from the bloodstream and is widely used for the treatment of PQ intoxication, the efficiency of HP in severe PQ poisoning has been disappointing [7]–[9]. On the contrary, in a series of studies using a canine model of PQ poisoning, Tominack and Pond et al [10]–[12] recommended that charcoal HP is effective and should be continuously administered for 6−8 h only if it can be initiated within 2 h of PQ injection [10], [11] or within 4 h of PQ ingestion [12]. However, no previous investigation has confirmed this finding in humans, and whether early HP improves the survival rate of severely PQ-poisoned patients remains uncertain.

To determine whether early HP improves the survival of patients with severe PQ poisoning, we performed a 10-year retrospective study to analyze PQ-poisoned patients admitted to Lin-Kou and Keelung Chang Gung Memorial Hospitals (CGMH) in Taiwan.

## Methods

### Ethics Statement

This clinical study was conducted in accordance with the Declaration of Helsinki and was approved by the Medical Ethics Committee of CGMH, a te*rtiary referral medical center* in Taiwan. Since this study involved retrospective review of existing data, Institutional Review Board approval was obtained, but without specific informed consent from patients. However, informed consent regarding the risk of acute PQ poisoning and all treatment modalities (including cardiopulmonary cerebral resuscitation, etc.) was obtained from all patients upon their initial admission. In addition, all individual information was securely protected (by delinking identifying information from the main dataset) and only available to the investigators. All data were analyzed anonymously. The Institutional Review Board of CGMH specifically waived the need for written informed consent.

### Patients

We retrospectively reviewed the medical charts of all patients with acute PQ poisoning who were admitted from January 1, 2000, to December 31, 2009. The selection of cases was based on the patients’ diagnosis on discharge and was accomplished through analysis of all of the medical records of the patients hospitalized in our department of internal medicine for this 10-year period. Thus, PQ-poisoned patients who arrived at the emergency room (ER) within 24 h after ingestion and had a dark blue colored urine PQ test result were included in this study. Patients with PQ poisoning were excluded from this study if they were <18 years of age, arrived at the ER within 24 h after ingestion but had a colorless or light blue colored urine PQ test result, arrived at the ER more than 24 h after ingestion, did not have urine PQ tests within 24 h after ingestion, did not have oral ingestion of PQ, joined the previous prospective study [13], or if they did not require admission to the wards and were discharged from the ER. Urine samples of all patients who arrived at the ER within 24 h of ingestion of 24% liquid PQ concentrate were measured using a UV spectrophotometer (DU-70; Beckman, Brea, CA, USA).

Two physicians who did not know the aim of this investigation participated in the study to abstract the charts using a standardized data collection form in a Microsoft Excel spreadsheet. The abstractors were trained in data abstraction by the principal investigator. Inter-rater reliability was calculated using 60 (6 per year) medical charts. Both abstractors reviewed the entire set of randomly selected medical charts. Inter-rater agreement was assessed using κ analysis. The inter-rater reliability was assessed after finalization of the medical record abstraction.

### Treatment Protocols

To prevent absorption of PQ in the gastrointestinal tract, the PQ-poisoned patients were administrated with 1 g/kg activated charcoal added to 250 mL of magnesium citrate through the nasogastric tubes following gastric lavage with normal saline in the ER. All patients also received 2 courses of 8-h active charcoal-containing HP therapy with a 4-h interval in the HD center or intensive care unit (ICU). HP was administered through 2 femoral venous catheters at a blood flow rate of 200 mL/min. An Adsorba 300C HP membrane (Gambro Dialysatoren GmbH Co., KG Hechingen, Germany) with a 300 m^2^ surface area was used, comprised of polypropylene housing material, activated charcoal adsorbent, and cellulose.

After HP therapy, patients who were admitted from January 1, 2000, to December 31, 2001, at Lin-Kou CGMH and from January 1, 2000, to December 31, 2003, at Keelung CGMH received high-dose therapy with 100 mg/day oral CP and 15 mg/day intravenous DX for 2 weeks. Based on a novel anti-inflammatory method for treating PQ-poisoned patients [14]–[16], patients who were admitted from January 1, 2002, to December 31, 2009, at Lin-Kou CGMH and from January 1, 2004, to December 31, 2009, at Keelung CGMH received repeated pulse therapy with intravenous methylprednisolone (MP; 1 g/day for 3 days) and CP (15 mg/kg/day for 2 days) initially, followed by 20 mg/day DX until the partial pressure of oxygen in arterial blood (PaO2) was >80 mmHg; MP was repeated (1 g/day for 3 days) and/or CP (15 mg/kg/day for 1 day) therapy was administered if PaO2 was <60 mmHg [14]–[16]. Arterial blood gas analysis, blood cell count, serum creatinine, and liver function tests were also performed.

### Outcome Measurement

We assessed the survival of PQ-poisoned patients and stratified the patients according to the timing of HP and the 2 different therapeutic methods, high-dose therapy and repeated pulse therapy. The survival time was calculated from the time of PQ ingestion to the time of death. The outcome measurement was patient mortality. Each surviving patient was followed for at least 60 days at wards or outpatient departments. The data were retrospectively collected by doctors who were not the authors and were unfamiliar with the study objectives throughout the duration of the research to avoid bias.

### Definitions

According to the results of previous animal studies [10]–[12], we defined early HP as initial HP therapy <4 h after PQ ingestion and late HP as initial HP ≥4 h after PQ ingestion. This is also the practice guideline of our hospital. In addition, we evaluated the associations between initial HP <5 h (including <4 h), initial HP <6 h (including <4 and <5 h) and initial HP <7 h (including <4, <5, and <6 h) after ingestion of PQ and the outcomes. Acute kidney injury was diagnosed according to the RIFLE classification (class R, class I, or class F). Patients who met any of the criteria of the RIFLE classification were classified as having acute kidney injury [17], [18]. Acute hepatitis was diagnosed when serum alanine aminotransferase (ALT) values exceeded >1.16 µkat/L (70 units/L; normal value <35 units/L) in patients who previously had normal liver function. Acute hypoxemia was diagnosed if a patient had PaO_2_<10.1 kPa (70 mmHg) by arterial blood gas analysis at room air.

### Statistical Analysis

Initially, the Kolmogorov-Smirnov test was applied for the distribution of the continuous variables. Unless otherwise stated, continuous variables are expressed as median (minimum, maximum) and categorical variables are expressed as numbers or percentages for each item. To compare the differences between the study groups, the Chi-square test with Fisher’s exact test was employed for categorical variables and the Mann-Whitney *U* test was applied to detect significant differences among non-normally distributed variables. To assess the relationship between treatment protocols and mortality, the Kaplan-Meier survival curves were compared with the log-rank test. The multivariate Cox proportional hazard model was used to measure all basal variables and to determine the potential variables for predicting mortality. The hazard ratio (HR) of death and 95% confidence interval (CI) were obtained. Further, forward stepwise analysis of the multivariate Cox proportional hazard model was used to evaluate the significant variables associated with HR for mortality. All study patients were also stratified into the repeated pulse group and high dose group to clarify whether different treatment methods influenced the outcome of early HP and late HP. The level of significance was set at *P*<0.05. The data were analyzed using SPSS, version 12.0 for Windows 95 (SPSS, Inc., Chicago, IL, USA).

## Results

**Figure 1:**
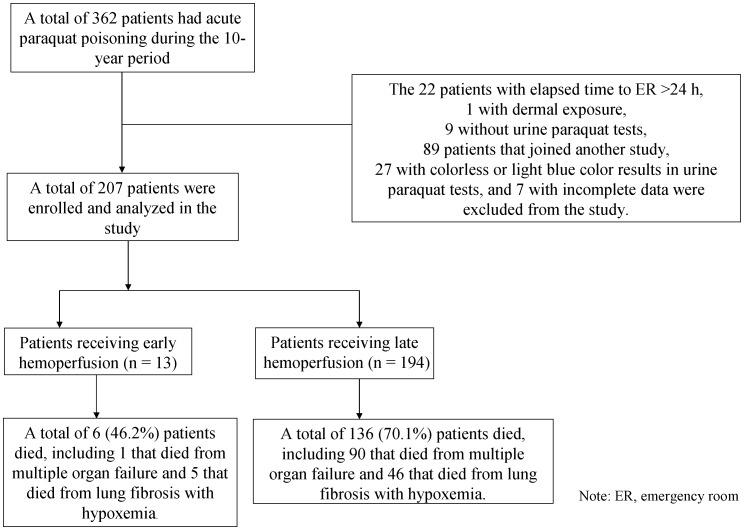
Overview of the study design, showing the enrollment and status of the patients.

Of 342 patients with PQ poisoning during the 10-year period, a total of 207 patients met the criteria and were included in this investigation (Figure 1). The inter-rater score for categorical variables was κ = 0.946, expressing a satisfactory inter-rater reliability.

**Figure 2:**
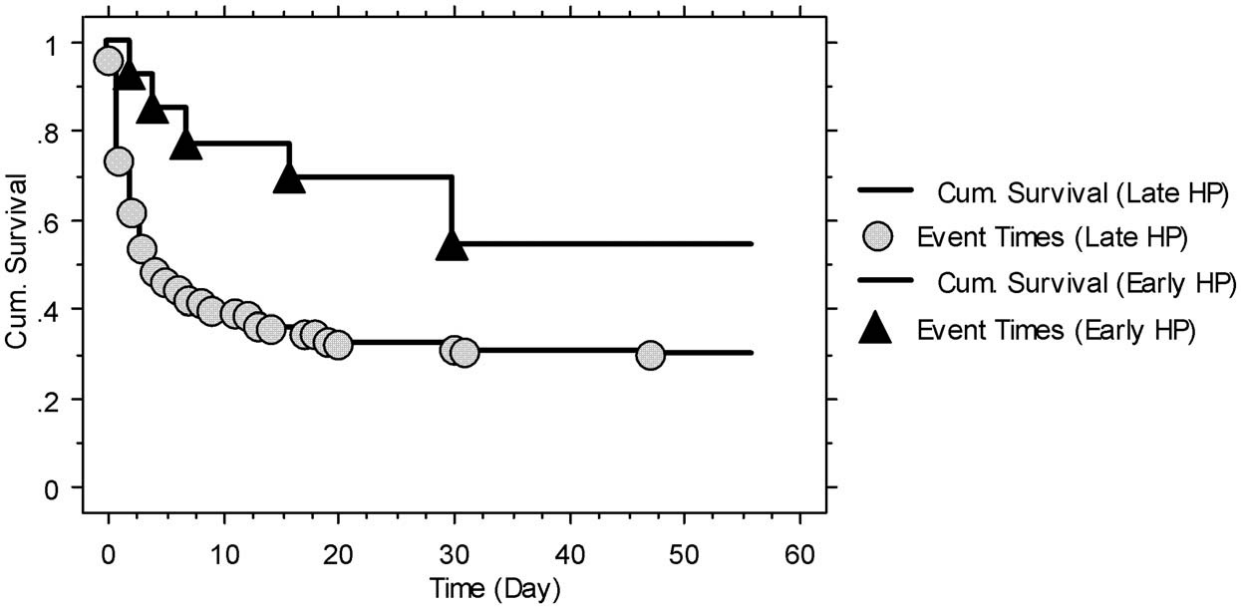
Kaplan-Meier survival analysis of severely paraquat-poisoned patients that received early hemoperfusion (<4 h; n = 13, 7/13 = 53.8%) and those that received late hemoperfusion (≥4 h; n = 194; 65/194 = 29.9%). Log-rank tests, Chi-square = 4.17; P = 0.041.

All patients were suicidal cases that had ingested a 24% liquid PQ concentrate. The mean age was 39.3±16.1 years and the median age was 36 years (minimum: 18 years; maximum: 79 years); most of the patients were young and healthy. The total time elapsed from the ingestion of PQ to arrival at the ER was <1 h in 3 (1.4%) patients, <2 h in 34 (16.4%) patients, <3 h in 72 (34.8%) patients, <4 h in 107 (51.7%) patients, and <5 h in 133 (64.3%) patients. All patients received gastric lavage and active charcoal >1 h after ingestion of PQ, whereas only 2 patients received these therapies within 2 h, and 23 patients received these therapies within 3 h. Of the 207 patients, 142 (68.6%) died within 60 days after exposure. Ninety-one (44.0%) patients died of multiple organ failure, including 52 (25.1%) that died within 24 h after ingestion. Fifty-one (24.6%) patients died of lung fibrosis with severe hypoxemia. Six of 13 (46.2%) patients that received HP <4 h after exposure died and 26 of 42 (61.9%) patients that received HP <5 h after exposure died, including the group of patients that received HP <4 h. Table 1 lists the baseline data of the survivors (n = 65) and non-survivors (n = 142). The survivors were younger and had a higher percentage of comorbidities and repeated pulse therapy, a decreased percentage of dark blue color in urine PQ tests, initial acute kidney injury, and lower baseline serum creatinine concentrations than the non-survivors. By Kaplan-Meier survival analysis, the survival rate of the early HP (<4 h) group was higher than that of the late HP (≥4 h) group (log-rank tests, *P = *0.041; Figure 2).

**Table 1:** Baseline characteristics of survivors and non-survivors among severely PQ-poisoned patients (n = 207).

Characteristics	Survivors(n = 65)	Non-survivors(n = 142)	*P*
Age (years)*	28 (18, 61)	42 (18, 79)	<0.001
Female sex	21 (32.3%)	30 (21.1%)	0.117
Body mass index (kg/m^2^)*	21.4 (14.3, 31.2)	21.3 (13.3, 30.7)	0.489
Smoking (Yes)	8 (12.3%)	22 (15.5%)	0.672
Alcoholism (Yes)	3 (4.6%)	2 (1.4%)	0.180
Comorbidities (Yes)	6 (9.2%)	3 (2.1%)	0.029
Time elapsed to ER (h)*	4.0 (0.5, 20.0)	3.8 (0.5, 21.0)	0.755
Time elapsed to PQ(U) test (h)*	4.5 (1.0, 21.0)	4.3 (1.0, 23.0)	0.958
Urine PQ tests-dark blue (Yes)	49 (75.4%)	138 (97.2%)	<0.001
Time elapsed to gastric lavage and active charcoal (h)*	5.8 (2.5, 21.8)	5.5 (2.3, 23.0)	0.906
Serum creatinine (mg/dL)*	0.8 (0.4, 2.4)	1.2(0.4, 7.0)	<0.001
Initial acute kidney injury (Yes)*	2 (3.1%)	48 (33.8%)	<0.001
Basal serum ALT (U/L)*	33 (6.0, 224.0)	31.0 (9.0, 462.0)	0.906
Initial acute hepatitis (Yes)	13 (20.0%)	37 (26.1%)	0.719
PaO2 (mmHg)*	93.4 (58.8, 121.4)	84.3 (32.9, 166.2)	0.054
Initial acute hypoxemia (Yes)	11 (16.9%)	29 (20.4%)	0.705
Repeated pulse therapy (Yes)	51 (78.5%)	85 (59.9%)	0.011
Early HP <4 h	7 (10.8%)	6 (4.2%)	0.118
Early HP <5 h	16 (24.6%)	26 (18.3%)	0.352

Note: The definitions of acute kidney injury, acute hepatitis, and hypoxemia are described in the Methods section. Comorbidities included 4 with hypertension, 2 with diabetes, and 3 with viral hepatitis. ALT: alanine aminotransferase; ER: emergency room; HP: hemoperfusion; PaO2: oxygen concentration in an arterial blood gas analysis, at room air; PQ: paraquat; PQ(U): urine paraquat.

* Data presented as median (minimum, maximum) and were assessed using the Mann-Whitney *U* test.

All baseline data were evaluated by multivariate Cox analysis. Age, urine PQ concentrations, repeated pulse therapy, initial acute kidney injury, and elapsed time to HP <4 h (HR = 0.39, 95% CI: 0.16–0.93; *P = *0.034) were significant variables associated with mortality in severely PQ-poisoned patients. Further forward stepwise analysis demonstrated that elapsed time to HP <4 h (HR = 0.38, 95% CI: 0.16–0.86; *P = *0.020) was a significant protective factor that reduced mortality in these patients, after adjustment for significantly related variables (Table 2). Similarly, when the elapsed time to HP <5 h was used as a predictor in multivariate Cox analysis, age, urine PQ concentrations, repeated pulse therapy, initial acute kidney injury, and elapsed time to HP <5 h (HR = 0.59, 95% CI: 0.35–1.00; *P = *0.048) were significant variables associated with mortality in severely PQ-poisoned patients. Forward stepwise analysis demonstrated that elapsed time to HP <5 h (HR = 0.60, 95% CI: 0.39–0.92; *P = *0.019) was also a significant protective factor that reduced mortality (Table 3), after adjustment for significantly related variables. However, neither elapsed time to HP <6 h nor <7 h after ingestion of PQ was a significant predictive factor of mortality in severely PQ-poisoned patients (data not shown).

**Table 2:** Forward stepwise multivariate Cox regression analysis for hazard ratios of all-cause mortality in severely PQ-poisoned patients, according to baseline variables and HP <4 h (n = 207).

Potential variable	Multivariate Cox hazard analysis	
	Hazard ratio (95% CI)	*P*
Age (years) (Each increase of 1 year)	1.02 (1.01−1.03)	<0.001
PQ(U)-navy blue color (Yes)	0.16 (0.06−0.43)	<0.001
Time elapsed to HP <4 h (Yes)	0.38 (0.16−0.86)	0.020
Initial acute kidney injury (Yes)	2.71 (1.82−4.05)	<0.001
Repeated pulse therapy (Yes)	0.63 (0.44−0.90)	0.011

CI: confidence interval; HP: hemoperfusion; PQ: paraquat; PQ(U): urine paraquat.

**Table 3:** Forward stepwise multivariate Cox regression analysis for hazard ratios of all-cause mortality in severely PQ-poisoned patients, according to baseline variables and HP <5 h (n = 207).

Potential variable	Multivariate Cox hazard analysis	
	Hazard ratio (95% CI)	*P*
Age (years) (Each increase of 1 year)	1.02 (1.01−1.03)	<0.001
PQ(U)-navy blue color (Yes)	0.15 (0.05−0.40)	<0.001
Time elapsed to HP <5 h (Yes)	0.60 (0.39−0.92)	0.019
Initial acute kidney injury (Yes)	2.62 (1.77−3.89)	<0.001
Repeated pulse therapy (Yes)	0.57 (0.40−0.82)	0.002

CI: confidence interval; HP: hemoperfusion; PQ: paraquat; PQ(U): urine paraquat.

All patients were stratified according to the 2 different treatment methods. Out of the 71 patients that received high-dose CP and DX therapy, a total of 57 (80.3%) patients died. Four of 5 (80%) patients receiving HP <4 h after exposure died and 17 of 21 (81%) patients receiving HP <5 h after exposure died. Elapsed times to HP <4 h (HR = 0.99, 95% CI: 0.31–3.18; *P = *0.992) and HP <5 h (HR = 0.68, 95% CI: 0.33–1.37; *P = *0.279) were not significant variables for predicting mortality in these patients by multivariate Cox regression analysis or subsequent forward stepwise analysis.

**Figure 3:**
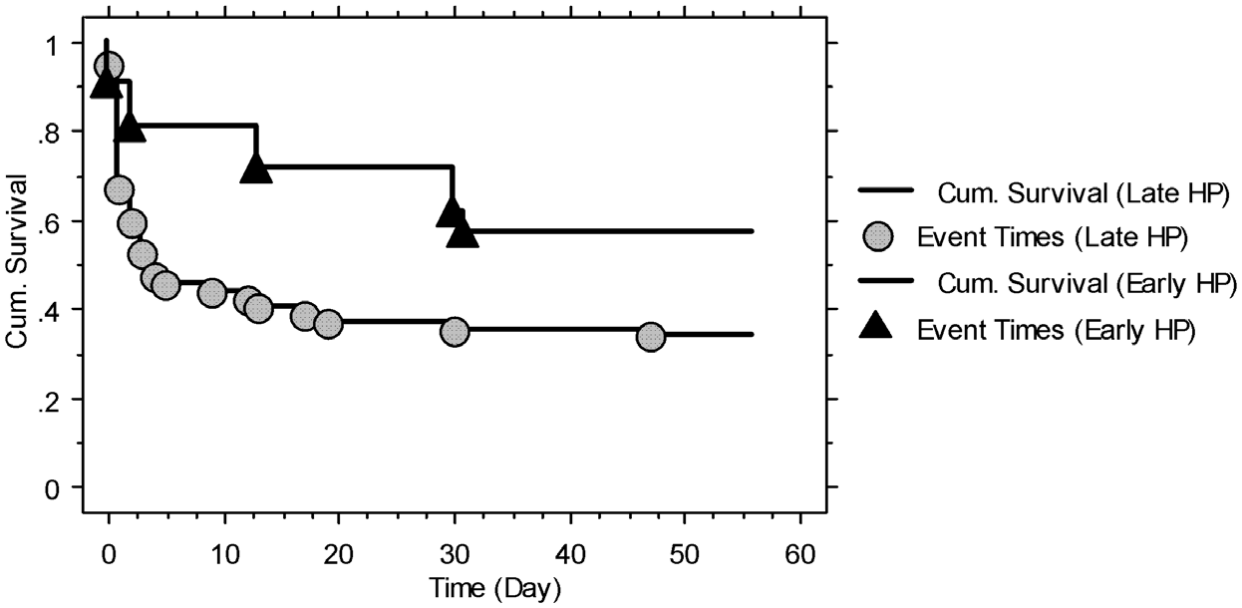
Kaplan-Meier survival analysis of severely paraquat-poisoned patients who received repeated pulse therapy with early hemoperfusion (<5 h; n = 21, 12/21 = 57.1%) and those that received late hemoperfusion (≥5 h; n = 115, 39/115 = 33.9%). Log-rank tests, Chi-square = 4.47; P = 0.035.

In contrast, 136 patients received repeated pulse MP and CP therapy, and a total of 85 (62.5%) patients died. There were 0 patients (0%) that arrived at the ER <1 h, 13 (9.6%) <2 h, 41 (30.1%) <3 h, 63 (46.3%) <4 h, and 84 (61.8%) <5 h after ingestion of PQ. Two of 8 (25%) patients that received HP <4 h after exposure died and 9 of 21 (42.9%) patients that received HP <5 h after exposure died. Table 4 lists the baseline data of the survivors (n = 51) and non-survivors (n = 85) who received repeated pulse therapy. The survivors were younger and had fewer dark blue colored urine PQ tests, initial acute kidney injury, and lower baseline serum creatinine concentrations, and a higher percentage of these patients received HP <4 h and <5 h than those of the non-survivors. By Kaplan-Meier survival analysis, the survival rate of the early HP (<5 h) group was higher than that of the late HP (≥5 h) group in severely PQ-poisoned patients treated with repeated pulse therapy (log-rank tests, *P = *0.035; Figure 3).

**Table 4:** Baseline characteristics of survivors and non-survivors among severely PQ-poisoned patients treated with repeated pulse therapy (n = 136).

Characteristics	Survivors(n = 51)	Non-survivors(n = 85)	*P*
Age (years)*	28 (18, 61)	43 (18, 79)	<0.001
Female sex	16 (32.3%)	13 (21.1%)	0.032
Body mass index (kg/m^2^)*	21.3 (14.3, 31.2)	21.4 (15.0, 30.7)	0.323
Smoking (Yes)	6 (12.3%)	12 (15.5%)	0.798
Alcoholism (Yes)	2 (4.6%)	1 (1.4%)	0.556
Comorbidities (Yes)	3 (9.2%)	2 (2.1%)	0.363
Time elapsed to ER (h)*	4.4 (1.0, 9.0)	4.0 (1.0, 21.0)	0.833
Time elapsed to PQ(U) test (h)*	4.8 (1.3, 10.0)	4.5 (1.8, 21.5)	0.880
Urine PQ tests-dark blue (Yes)	42 (75.4%)	83 (97.2%)	0.002
Time elapsed to gastric lavage and active charcoal (h)*	5.3 (1.5, 9.8)	4.8 (1.8, 21.8)	0.846
Serum creatinine (mg/dL)*	0.8 (0.4, 1.5)	1.2 (0.5, 3.3)	<0.001
Initial acute kidney injury (Yes)	1 (3.1%)	28 (33.8%)	<0.001
Basal serum ALT (U/L)*	33.0 (6.0, 224.0)	27.0 (9.0, 462.0)	0.188
Initial acute hepatitis (Yes)	8 (20.0%)	19 (26.1%)	0.383
PaO2 (mmHg)*	92.0 (61.2, 109.5)	84.7 (32.9, 166.2)	0.173
Initial acute hypoxemia (Yes)	10 (16.9%)	20(20.4%)	0.705
Early HP <4 h	6 (10.8%)	2 (4.2%)	0.052
Early HP <5 h	12 (24.6%)	9 (18.3%)	0.052

Note: The definitions of acute kidney injury, acute hepatitis, and hypoxemia are described in the Methods section. Comorbidities included 4 with hypertension, 2 with diabetes, and 3 with viral hepatitis. ALT: alanine aminotransferase; ER: emergency room; HP: hemoperfusion; PaO2: oxygen concentration in an arterial blood gas analysis, at room air; PQ: paraquat; PQ(U): urine paraquat.

* Data presented as median (minimum, maximum) and were assessed by Mann-Whitney *U* tests.

Multivariate Cox analysis showed that age, initial acute kidney injury, and elapsed time to HP <4 h after PQ ingestion (HR = 0.18, 95% CI: 0.04–0.78; *P = *0.023) were significant variables associated with mortality in severely PQ-poisoned patients treated with repeated pulse therapy. Further forward stepwise analysis demonstrated that elapsed time to HP <4 h (HR = 0.19, 95% CI: 0.05–0.79; *P = *0.022) was a significant protective factor that reduced mortality in these patients, after adjustment for significantly related variables (Table 5). Similarly, when the elapsed time to HP <5 h was used as a predictor in multivariate Cox analysis, age, initial acute kidney injury, and elapsed time to HP <5 h after PQ ingestion (HR = 0.39, 95% CI: 0.17–0.87; *P = *0.022) were significant variables associated with mortality in severely PQ-poisoned patients treated with repeated pulse therapy. Forward stepwise analysis demonstrated that the elapsed time to HP <5 h (HR = 0.49, 95% CI: 0.24–0.98; *P = *0.043) was also a significant protective factor leading to reduced mortality (Table 6), after adjustment for significantly related variables. However, elapsed time to HP <6 and <7 h after ingestion of PQ were not significant factors for predicting mortality in these patients (data not shown).

**Table 5:** Forward stepwise multivariate Cox regression analysis for hazard ratios of mortality in severely PQ-poisoned patients treated with repeated pulse therapy, according to baseline variables and HP <4 h (n = 136).

Potential variable	Multivariate Cox hazard analysis	
	Hazard ratio (95% CI)	*P*
Age (years) (Each increase of 1 year)	1.03 (1.01−1.04)	<0.001
Time elapsed to HP <4 h (Yes)	0.19 (0.05−0.79)	0.022
Initial acute kidney injury (Yes)	2.84 (1.68−4.78)	<0.001

CI: confidence interval; HP: hemoperfusion; PQ: paraquat.

**Table 6:** Forward stepwise multivariate Cox regression analysis for hazard ratios of mortality in severely PQ-poisoned patients treated with repeated pulse therapy, according to baseline variables and HP <5 h after PQ ingestion (n = 136).

Potential variable	Multivariate Cox hazard analysis	
	Hazard ratio (95% CI)	*P*
Age (years) (Each increase of 1 year)	1.03 (1.01−1.04)	<0.001
Time elapsed to HP <5 h (Yes)	0.49 (0.24−0.98)	0.043
Initial acute kidney injury (Yes)	2.49 (1.48−4.18)	0.001

CI: confidence interval; HP: hemoperfusion; PQ: paraquat.

## Discussion

The results of the present study revealed (after adjustment for significantly related variables) that the time that elapsed from exposure to HP <4 h or <5 h was associated with a 62% and 41% reduction of the relative risk of mortality of all severely PQ-poisoned patients, respectively. Further analysis demonstrated that early HP reduced the risk of mortality only in patients treated with repeated pulse therapy, not in those treated with high-dose therapy. The time elapsed from exposure to HP <4.0 h or <5.0 h may significantly reduce the relative risk of mortality by approximately 81% and 51% in severely PQ-poisoned patients treated with repeated pulse therapy, respectively. The time elapsed to administration of gastric lavage and active charcoal (HR = 1.06, 95% CI: 0.65–1.72; *P = *0.802), <3 h (n = 23; HR = 1.15, 95% CI: 0.59–2.24; *P = *0.691), and <4 h (n = 46; HR = 1.25, 95% CI: 0.74–2.10; *P = *0.404, data not shown) were not significantly associated with the HR of mortality by multivariate Cox analysis; hence, early HP was associated with reduced risk for mortality in these patients. For the first time, the findings of this human study confirm the results of previous studies in canine models [10]–[12], which demonstrated that HP administered within 4 h after ingestion effectively reduced the mortality of animals. These results are also supported by another animal investigation, which indicated that early HP was effective in treating PQ poisoning and improved the survival rate even in severely poisoned pigs [19]. Ten pigs were orally treated with PQ at a dose of 70 mg/kg of body weight, a lethal dose for all pigs, and received HP 2 h after ingestion for 6 h (n = 4). Prolonged 6-h HP successfully rescued 3 out of 4 intoxicated pigs. However, in a study of PQ-poisoned patients, Suzuki et al [20] showed that aggressive HP (defined as HP administered for ≥10 h during the first 24 h after ingestion) did not improve the outcome but did improve the survival time, relative to HP administered for <10 h during the initial 24 h after ingestion. Moreover, previous studies also reported that the survival rate of PQ-poisoned patients was not improved, despite the fact that PQ can be removed effectively from the plasma by HP in humans [1]–[3]. The reason for this may be that when HP is initiated, potentially lethal concentrations of PQ have already been attained via active transport in the highly vascularized tissues of the vital organs and in pneumocytes [10]–[12], [21], [22]. The peak time of plasma PQ is 1−3 h, that of lung cells is approximately 4−5 h, and nearly 90% of PQ disappears 5−6 h later in the plasma after ingestion [6], [22]. Therefore, patients that received HP therapy as early as possible after PQ ingestion within the therapeutic window would have a significant amount of PQ removed from the blood and might indirectly reduce the amount of PQ accumulated in the lung cells. To our knowledge, this is the first report describing these predictive factors for reduced mortality in PQ-poisoned patients. Based on these findings, it seems reasonable to suggest that proper timing is critical, particularly in the management of severely PQ-poisoned patients. In Taiwan, the government educates the population that PQ misuse is dangerous and fatal. Individuals should be sent to the hospitals with facilities to manage PQ intoxication as soon as possible if PQ exposure is suspected. In our hospital, the urine dithionite test is available at all times, and the medical dialysis staff is informed of the results in 10 minutes. If we could shorten the time elapsed from when patients present for medical attention to the diagnosis of PQ exposure, it would be possible to perform early HP and more severely PQ-poisoned patients might be saved.

The study results also indicated that shortening the time elapsed from ER arrival to HP administration might be crucial for reducing the mortality rates of severely PQ-poisoned patients with repeated pulse therapy. Although 46.3% (63/136) of patients arrived at the ER within 4 h after ingestion of PQ, only 5.9% (8/136) of patients received HP within 4 h after exposure. Similarly, 61.8% (84/136) of patients arrived at the ER within 5 h after ingestion of PQ, while only 15.4% (21/136) of patients received HP within 5 h after exposure. The median time elapsed from arrival at the ER to the initiation of HP was 2.5 h (minimum: 2 h; maximum: 7 h). Because shortening the time elapsed from ER arrival to HP initiation may be important for rescuing these patients, an aggressive attitude of the medical staff is essential. In the ER, history taking, physical examination, gastric lavage, active charcoal, and PQ urine tests can quickly establish the diagnosis of PQ intoxication. When a diagnosis is made, the HP and ICU staff should be notified and the patients should be sent to the ICU for HP therapy immediately. Furthermore, performing HP in the ER may shorten the elapsed time from ER arrival to HP treatment. Continuous arteriovenous HP (CAVHP), a pump-less HP technique first developed by our research team in 1992 and reported in 1993 [23], [24], can easily be performed in the ER [25] immediately. If no machine is available for HP, CAVHP could be administered in the ER or ICU [25]. If the time elapsed from ER arrival to initiation of HP was reduced to <1 h, 46.3% (63/136) to 61.8% (84/136) of patients would have received HP within 5 h after ingestion of PQ, and the survival rate might have increased. However, only 5.9% (8/136) to 15.4% (21/136) of patients received HP <4 h to <5 h after PQ exposure in the current study. Indeed, further research is needed to confirm this observation.

Our study also demonstrated that after the significantly related variables were adjusted, repeated pulse therapy was associated with 37% (HP <4 h) and 43% (HP <5 h) reductions in the relative risk of mortality in all severely PQ-poisoned patients, compared to high-dose therapy. These results are similar to recent reports assessing the effect of pulse therapy in patients with PQ poisoning [13], [26]. Of note, the patients that received high-dose therapy had a mortality rate of 80.3% (57/71), similar to those of previous reports [3]–[5]. Furthermore, elapsed time to HP <4 and <5 h were associated with 81% and 51% reduction in the relative risk of mortality in severely PQ-poisoned patients treated with repeated pulse therapy, respectively. The observation of early HP improving survival in patients treated with repeated pulse therapy, but not in patients with high-dose CP and DX therapy might be explained by several possible reasons. First, the strong anti-inflammatory effect of mega-dose MP and CP-induced leucopenia and its immunosuppressive effects [4], [17] may play a role in reduction of lung fibrosis and inflammation [27]. Second, prolonged DX treatment may attenuate the inflammatory changes of the lung. Importantly, recent rodent studies [28], [29] demonstrated that the administration of a mega-dose of DX, similar to MP pulse therapy, decreased the PQ accumulation in lung cells to approximately 40% and improved tissue healing as a result of the induction of P-glycoprotein synthesis. However, further study is needed to clarify these hypotheses.

Some limitations of this investigation should be noted. First, the long time frame of the study (10 years) is a limitation, as ICU supportive treatment protocols underwent important changes in this period. Second, the study was retrospective and performed in a single center. Although this study was a retrospective investigation and observational bias is always possible when reviewing charts, the reasonably high inter-rater reliability suggests that the study did not result in substantial investigator bias. Third, while the study indicated the early HP may effectively improve the survival of severely PQ-poisoned patients, further prospective study with a large group of early HP patients is required to confirm our observation due to the small number of patients with early HP that were enrolled. Fourth, the 2 very different supportive pharmacological treatments may be a limitation in the clinical setting. Finally, since only some of study patients had serum PQ concentrations but all patients had urine PQ tests, we could only analyze the patients with urine PQ tests in the current study. However, plasma PQ concentrations drop rapidly after exposure. A previous report [30] indicated that an error of 1−2 h in the estimation of the time of ingestion could shift the survival curve of a patient from 30% to 70%. Practical experience suggests that such estimation errors are not uncommon [28]. Since previous studies [3]–[5] have shown that urine tests within the first 24 h of intoxication are good predictors of the outcome and prognosis, the sodium dithionite test was a reasonable indicator of the severity of PQ intoxication in our patients.

In conclusion, the present study first demonstrates that, similar to previous animal studies [10]–[12], early HP (<4 or <5 h after ingestion of PQ) is associated with decreased mortality in severely PQ-poisoned patients after adjusting for significantly related variables. However, further studies are needed to confirm our observations.
